# Hemostasis in Pre-Eclamptic Women and Their Offspring: Current Knowledge and Hemostasis Assessment with Viscoelastic Tests

**DOI:** 10.3390/diagnostics14030347

**Published:** 2024-02-05

**Authors:** Christos-Georgios Kontovazainitis, Dimitra Gialamprinou, Theodoros Theodoridis, Georgios Mitsiakos

**Affiliations:** 1 2nd Neonatal Department and Neonatal Intensive Care Unit (NICU), “Papageorgiou” University Hospital, Aristotle University of Thessaloniki, 56403 Thessaloniki, Greece; chgkontovazainitis@gmail.com (C.-G.K.); gialamprinou@gmail.com (D.G.); 2 1st Department of Obstetrics and Gynecology, “Papageorgiou” University Hospital, Aristotle University of Thessaloniki, 56403 Thessaloniki, Greece; theodtheo@yahoo.gr

**Keywords:** blood coagulation, hemostasis, infant, newborn, pre-eclampsia, pregnancy, thromboelastography, thromboelastometry

## Abstract

Pre-eclampsia (PE) is a placenta-mediated disease and remains a major cause of maternal and neonatal mortality and morbidity. As PE develops, normal pregnancy’s hypercoagulable balance is disrupted, leading to platelet hyperactivation, excessive pathological hypercoagulability, and perturbed fibrinolysis. This narrative review aims to summarize the current knowledge regarding hemostasis in PE compared with healthy gestation and the potential effects of maternal PE on neonatal hemostasis. Finally, it aims to discuss hemostasis assessments for normal pregnancies and PE, emphasizing the role of viscoelastic tests, namely, thromboelastography (TEG) and thromboelastometry (ROTEM), for monitoring PE-associated hemostatic alterations. The use of TEG/ROTEM for assessing the hemostatic profile of PE women has been little considered, even though conventional coagulation tests (CCTs) have not helped to monitor hemostasis in this population. Compared with normal pregnancy, TEG/ROTEM in PE reveals an excessive hypercoagulability analogous with the severity of the disease, characterized by higher-stability fibrin clots. The TEG/ROTEM parameters can reflect PE severity and may be used for monitoring and as predictive markers for the disease.

## 1. Introduction

Pre-eclampsia (PE), a gestational hypertensive disorder, complicates 2–8% of pregnancies worldwide [1]. PE and eclampsia are the premier causes of maternal and neonatal mortality and morbidity, responsible for about 13% of maternal deaths in developed countries [2]. During early pregnancy, an abnormal trophoblastic invasion leading to abnormal implantation causes endothelial dysfunction, mediated by immunological and inflammatory factors. These events result in a hemostatic imbalance, reflected mainly by excessive hypercoagulability, hypertension, proteinuria, and other clinical manifestations [1,3,4].

Normal gestation is described as a procoagulant state. The uteroplacental unit’s hemostatic balance during healthy gestation must compensate for the physiological alterations occurring during pregnancy. The hemostatic system counterbalances the disruption of the maternal decidual vessels (a fact with a high concomitant risk of hemorrhage), which takes place during the trophoblastic invasion. Parallelly, throughout gestation, normal placenta perfusion must be maintained; an adequate and smooth blood flow must be conserved in an organ where the peripheral resistances need to be low. Finally, the hemostatic system must be balanced to overcome the significant blood loss that naturally occurs during delivery at the time of placental separation [3].

Thus, the above system is vulnerable to the endothelial activation and dysfunction that occurs in PE, which is characterized by platelet hyperactivation and hypercoagulation, with increased tissue factor (TF) expression and activity and fibrinolysis deregulation [5].

Conventional coagulation tests (CCTs) fail to reflect the hemostatic alterations that occur in PE patients adequately and cannot monitor the disease’s progress and severity [6,7]. Viscoelastic tests, namely, thromboelastography (TEG) and rotational thromboelastometry (ROTEM), which are rapid, easy-to-use bedside methods, are more capable of monitoring the above-mentioned alterations. They dynamically and universally examine, for the whole blood, the hemostasis phases from initiation through amplification and propagation to termination and fibrinolysis. Therefore, their use in PE patients and their neonates may be helpful [8,9,10]. However, there is a gap in the literature regarding their use, especially in the neonates of pre-eclamptic women.

This narrative review aims to summarize the current literature regarding the hemostatic profile of pregnant women with PE and their offspring compared with healthy pregnant women, emphasizing the use of viscoelastic tests for assessing their hemostatic profile. First, hemostasis in healthy pregnant women is described. Subsequently, the hemostatic profile of women with PE is discussed. Information about neonatal developmental hemostasis is provided afterward, and the potential effect of maternal PE on neonatal hemostasis is discussed. The use of CCTs for evaluating hemostasis in healthy pregnant women and PE patients is then mentioned. This review then focuses on using TEG and ROTEM for these populations and discusses the results from different studies that have assessed this subject. Finally, future insights about the utility of TEG and ROTEM in pregnant women, especially PE patients and their neonates, are considered.

## 2. Hemostasis in Healthy Pregnancies

Normal gestation is described as a procoagulant state, with the amniotic fluid being described as an activator of coagulation [11].

### 2.1. Coagulation and Fibrinolysis

Coagulation and fibrinolysis are enhanced during gestation but stay in equilibrium to preserve hemostatic balance [11].

Thrombin production progressively rises [12]. Most coagulation factor levels (VII, VIII, IX, and XII) are increased in normal pregnant women, excluding the fibrin-stabilizing factor XIII, which progressively declines throughout gestation [13,14]. There are controversies regarding factor XI levels, although they are probably reduced. Factor II, X, and V levels are within normal ranges, while the von Willebrand factor (vWF) antigen levels are five times higher than before pregnancy, compensating for the decrease in the platelet count. Fibrinogen (factor I) varies between 200 and 400 mg/dL in normal non-pregnant women, and its levels rise by around 50 percent throughout a healthy pregnancy. This fact is associated with the elevated erythrocyte sedimentation rate, which is noted in normal gestation [2].

There have been controversial results regarding fibrinolysis. It has been suggested that it is probably decreased. Thus, fibrin formation is essentially promoted, and increased fibrin deposits have been found in histological samples from the chorionic villous area of normal placentas [2,15]. However, it has also been noted that fibrinolysis may be enhanced during pregnancy. Elevated plasminogen levels counteract fibrin accumulation. Plasminogen is converted with the aid of tissue plasminogen activator (t-PA) into plasmin [14,16]. The urokinase-type plasminogen activator (u-PA) is equally increased [2]. Plasmin enhances fibrinolysis, resulting in elevated D-dimer levels [15]. Nevertheless, during gestation, the overall results favor the procoagulant condition [14,16].

During pregnancy, coagulation inhibitors’ levels are decreased. Antithrombin (AT) levels decrease by up to 13% compared with normal levels at full term, and they continue to fall by 30% from this threshold after delivery [2,17]. Heparin cofactor II (HCII) and tissue factor pathway inhibitor (TFPI) (which normally blocks the initiation of blood coagulation by inhibiting the TF–fVIIa complex) levels are increased [2]. As gestation progresses, protein S levels gradually decline by 60%, while activated protein C resistance increases, with a simultaneous 30% decline in its levels. Physiologically, the procoagulant activated factors Va and VIIIa are neutralized by the activated regulatory protein C with the aid of protein S, which acts as a cofactor [2,17]. Plasminogen activator inhibitor 1 (PAI-1) and placenta-derived PAI-2 levels rise four and five times, respectively, while it is disputable whether the thrombin-activatable fibrinolysis inhibitor (TAFI) levels remain stable or increase [2]. Note that the PAI-2 increase is proportional to the development of the placenta. The ratio of PAI-1/PAI-2 is decreased [3]. The expression of endothelial t-PA is downregulated due to the PAI-1 increase. Thus, t-PA activity is reduced, and the t-PA/PAI-1 ratio decreases [3]. The hypercoagulable markers, namely, the prothrombin fragments 1 + 2 (F1+2), Thrombin-AT (TAT) complexes, and D-dimers, are at levels similar to those seen in acute thrombotic events [3].

### 2.2. Platelets

During normal pregnancy, platelet counts are lower (up to 10%) and continue to fall throughout pregnancy, while this decline is more pronounced in twin pregnancies. The average platelet count returns to its normal values 1 to 3 months after delivery [2,18]. This gradual decrease is due to hemodilution, which physiologically occurs during gestation. Likewise, platelet consumption in the uteroplacental unit and aggregation is elevated, promoting an increased number of more immature and larger platelets [19,20,21]. Additionally, splenic enlargement, which naturally occurs in normal gestation, might contribute to earlier platelet destruction [22,23].

Whether platelet activity is increased or decreased during normal pregnancy is disputed. Based on flow cytometry findings, it may be moderately increased even in normal pregnancies, especially during the third trimester [2,22,23]. Increased platelet activity may be due to elevated calcium mobilization and decreased basal cAMP levels [2]. Platelet activation with a concomitant release of vasoactive substances occurs during the repair of the uteroplacental unit. The latter is linked with the pathogenesis of obstetric complications, such as pre-eclampsia [21].

## 3. Hemostasis in Pre-Eclampsia

Hemostasis in PE is characterized by enhanced thrombin generation with elevated inhibitor proteins, perturbed fibrinolytic activity, and perturbed fibrinolysis control mechanisms such as elevated PAI-1 and t-PA and reduced PAI-2 [24].

### 3.1. Genetic Factors

PE has a hereditary predisposition for developing the disease, with an estimated 50–55% heritability factor. Hence, women born to mothers with PE, as well as twins, present an increased risk for developing PE. The familial form of PE is associated with a more severe phenotype [25,26,27]. Genes implicated in endothelial function, lipid metabolism, oxidative stress, immune response, and thrombophilia have been associated with PE [27,28,29,30,31,32,33]. Environmental factors affect the phenotypic expression; thus, it will vary among similar genotypes [34].

Some polymorphisms strongly associated with PE concern the following genes: the angiotensinogen (AGT) gene, endothelial nitric oxide synthase (NOS3) gene, angiotensin-converting enzyme (ACE) gene, angiotensin II receptor type 1 (AGTR1) gene, cytotoxic T lymphocyte-associated (CTLA4) gene, lipoprotein lipase (LPL) gene, serine peptidase inhibitor (SERPINE1) gene, and the inhibitor of nuclear factor kappa B kinase gamma (IKBKG) gene [25,28,30,35,36,37].

Mutations in genes involved in the immune response are associated with the development of PE [25,28,30,35,36,37]. Regarding human leukocyte antigens (HLAs), HLA-G plays a significant role in maternal immune tolerance during gestation as it is expressed by placental extravillous trophoblasts. Maternal 1597ΔC mutation in the HLA-G gene, found more frequently in African-American women, reduces placental HLA-G expression, leading to immune maladaptation and abnormal placental vascularization. Thus, these women are prone to developing PE [38]. Single nucleotide polymorphisms (SNPs) in interferon-gamma (IFN-γ), tumor necrosis factor α (TNFa), and interleukin (IL) 4,6,10,17A, and 22 are associated with PE development and the disease’s severity [27].

It is essential to underline the importance of thrombophilia-associated genes in the disease’s development. Hypercoagulability characterizes the disease as it results in reduced placental blood perfusion due to microthrombi, consequently leading to placental ischemia. Thus, hypercoagulability-related genes enhance PE development, and women with hereditary thrombophilia are more prone to developing PE [39,40,41]. The following thrombophilia-associated SNPs have been associated with increased risk for PE: Leiden G1691A (factor V gene), 20210A (prothrombin gene), and C677T (methylene tetrahydrofolate reductase MTHFR gene) [42,43,44,45]. The presence of 20210A (prothrombin) and G1691A (factor V Leiden) SNPs are associated with elevated risk for PE (2-fold and 1.6-fold, respectively). Parallelly, these polymorphisms are associated with a greater risk of developing PE with severe features, at a rate of 3-fold in women with prothrombin 20210A polymorphism and 2.45-fold in women with factor V G1691A polymorphism [46].

### 3.2. Endothelial Dysfunction

**Figure 1:**
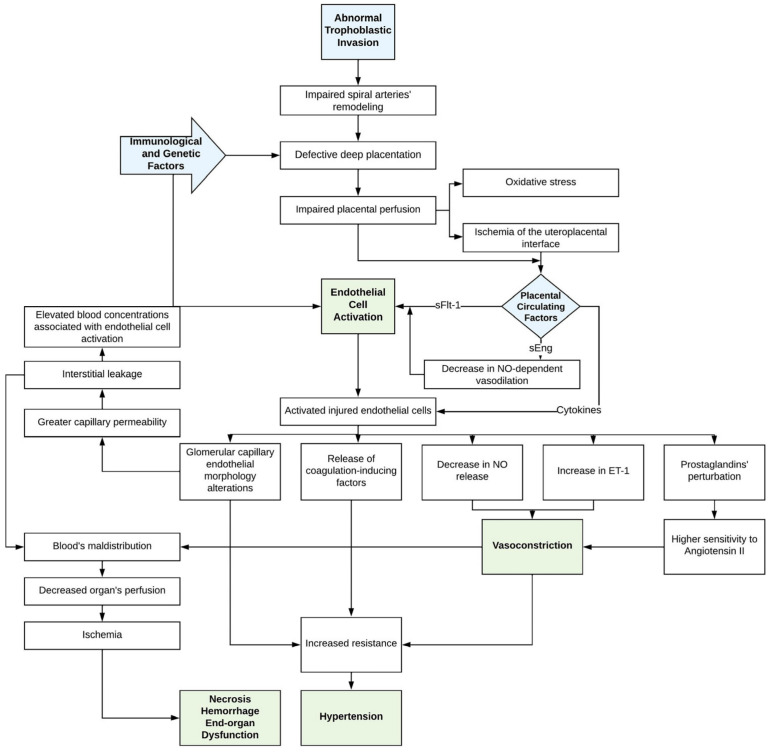
Pathogenesis of pre-eclampsia. sFlt-1: soluble fms-like tyrosine kinase 1; sEng: soluble endoglin; ET-1: endothelin 1; NO: nitric oxide.

PE endothelial dysfunction leads to the hemostatic imbalance that characterizes the disease. In early gestation, PE pathogenesis is initiated with abnormal trophoblastic invasion during the placenta development, leading to oxidative stress and uteroplacental bed ischemia. The above facts contribute to the release of anti-angiogenic factors, such as soluble endoglin (sEng) and soluble fms-like tyrosine kinase 1 (sFlt-1), restricting the nitric oxide-dependent (NO-dependent) vasodilation. Thus, endothelial cells are activated, leading to morphological capillary alterations and the reduction of NO. Cytokines, such as IL-2, IL-6, IL-8, and TNFa, are mediators in the above process. Subsequently, endothelin-1 (ET-1) is expressed and released, while the prostaglandin balance is disrupted. As a result, the sensitivity to vasoconstrictors is increased. The latter is induced by the aforementioned ET-1 overexpression, NO reduction, and prostaglandin imbalance. The vasoconstriction becomes more eminent, creating a “feed-forward loop”; endothelial injury results in intrinsic vascular factors’ and lipids’ release, which again jeopardizes the endothelial function. Therefore, endothelial dysfunction is established. Activated and injured endothelium releases TF and PAI-1, leading to microthrombosis and, thus, impaired vascular integrity. The release of coagulation-inducing factors further promotes vasopressors’ sensitivity and the subendothelial deposition of blood elements, such as platelets and fibrinogen, activating the hemostasis [47,48]. Figure 1 summarizes the pathogenesis of PE with an emphasis on endothelial dysfunction.

The role of endothelial progenitor cells (EPCs) in PE’s endothelial dysfunction is essential. Normally, EPCs contribute to endothelial cell homeostasis, vascular remodeling, and endothelial repair. EPCs are mobilized to the circulation by NO-dependent pathways in response to vascular endothelial growth factor (VEGF) and placental growth factor (PlGF) [49]. In PE, the number and function of EPCs is defective. The defective endothelial repair capacity in PE is reflected by decreased EPCs expressing CD34 or CD133 in combination with vascular endothelial growth factor receptor-2 (VEGFR-2) and CD45, CD14, CDParallelly, colony-forming units of maternal EPCs were likewise reduced in PE. EPCs and colony-forming unit counts did not correlate with plasma sFlt-1 or PlGF among normal pregnancies and women with PE [49,50].

### 3.3. Role of microRNAs in Endothelial Dysfunction and Hypercoagulation

MicroRNAs (miRNAs) play a role in endothelial dysfunction and hypercoagulation in PE by regulating trophoblast function, angiogenesis, and mesenchymal stem cell function [51,52]. It is known that miRNAs manage post-transcriptional regulation by causing mRNA destabilization and/or the inhibition of translation and play an important role in regulating placental development [53]. The expression of miRNAs targeting genes essential for trophoblast proliferation, differentiation, and migration is altered in PE. The latter leads to placental malformation [53,54,55]. For example, in PE, miR-515-5p (C19MC cluster) is elevated. miR-515-5p targets and inhibits cytochrome P450 CYP19A1 and glial cells missing transcription factor 1 GCM1 genes, which are essential in trophoblast differentiation [55]. In PE, the NODAL proteins’ pathway is disrupted by dysregulated miRNA expression, inhibiting trophoblast migration, invasion, and proliferation. The expressions of miR-378a-5p, which represses NODAL protein expression, and miR-376c, which targets activin receptor-like kinase 5 and 7 (ALK5 and ALK7) proteins of the NODAL pathway, are downregulated in women with PE [56,57]. Variants in the 3′UTR may alter gene expression by a loss or gain of miRNA recognition [53]. Some examples are the variants found in the 3′UTR of pentraxin-related protein 3 (PTX3; gene implicated in inflammation regulation), regulator of G-protein signaling 2 (RGS2; gene implicated in arterial pressure regulation), and MTHFR genes [58,59,60].

### 3.4. Hemostatic Imbalance

**Figure 2:**
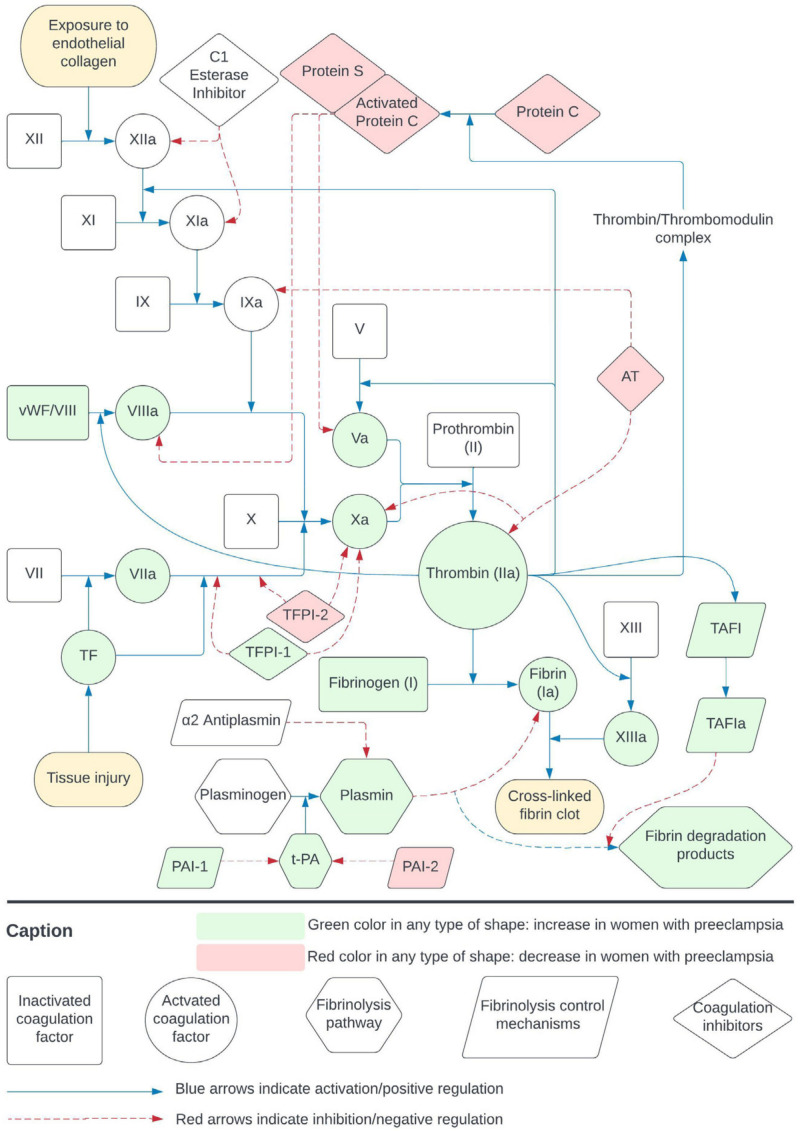
Coagulation cascade in women with pre-eclampsia compared with healthy pregnant individuals. The figure depicts the reported increase (with green color) or reduction (with red color) of the hemostasis parameters. Rectangle-shaped parameters represent inactivated coagulation factors, circle-shaped parameters represent activated coagulation factors, hexagon-shaped parameters represent proteins of the fibrinolysis pathway, parallelogram-shaped parameters represent fibrinolysis control mechanisms, and diamond-shaped parameters represent coagulation inhibitors. Blue arrows indicate activation and positive regulation, while red arrows indicate inhibition and negative regulation. TF: Tissue factor; AT: antithrombin; vWF: von Willebrand factor; TFPI-1,2: tissue factor pathway inhibitors 1,2; TAFI: thrombin activatable fibrinolysis inhibitor; TAFIa: active thrombin activatable fibrinolysis inhibitor; PAI-1,2: plasminogen activator inhibitors 1,2; t-PA: tissue plasminogen activator.

Hemostatic balance alterations resulting in significant uteroplacental thrombosis characterize PE. In PE, the endothelial dysfunction results in a hemostatic imbalance foremost and initially in the placenta. Microthrombi are formatted, placenta perfusion is further reduced, and fibrin deposits are created, forming placental infarction zones, which are eminent in PE patients. Syncytiotrophoblast microparticles originated de facto from the placenta, are increased in PE patients, and play a vital role in the inflammatory intravascular reactions and the concomitant hypercoagulability that characterizes PE. As it becomes systemic, the above process expands to other organs. Endothelial and mesenchymal damage, as well as fibrin deposits with fibroid necrosis, are found in the subendothelial space of renal tissue, leading to impaired renal function [2]. Compared with a normal healthy pregnancy, where there is a de facto hypercoagulable state, PE demonstrates a more excessive amount of coagulation activation and fibrinolysis deregulation [3]. Figure 2 summarizes the coagulation cascade, depicting the reported increase or reduction of hemostasis parameters in PE.

### 3.5. Coagulation and Fibrinolysis

Regarding coagulation factors in PE patients, their levels seem to remain within the normal pregnancy ranges, except for TF, which is found mostly elevated but for whom controversial data are extracted [2,3,61,62], and factor VIII, whose consumption is elevated. The levels of coagulation factors are expected to be affected in pregnant women with hereditary or acquired thrombophilias, who are more prone to developing PE [14,63]. Regarding fibrinogen, its levels are mostly higher, but not significantly from those noted in healthy pregnancies. vWF antigen levels and fibrinopeptides A and B are increased [2,3,7,64,65]. The endogenous thrombin potential, a marker that measures the combined effect of the multiple factors determining coagulation, is elevated, while the lag time of thrombin generation is shortened compared with healthy pregnant individuals [2,3].

Regarding fibrinolysis, PE is associated with higher PAI-1 and lower PAI-2 levels compared with healthy pregnancies. Higher PAI-1 levels are found early in gestations with placental dysfunction. As PAI-2 originates mainly from the placenta’s trophoblastic tissue, its lower levels are due to PE’s abnormal placental development. Considering the above, the PAI-1/PAI-2 ratio, which is used as a PE marker, is elevated [66,67]. Parallelly, t-PA mRNA expression in the placenta and t-PA systemic activity rise. D-dimers’ levels and TAT complexes in plasma tend to rise, but there are controversial findings on whether this rise is significant [2].

Coagulation inhibitors, such as protein C, protein S, and ATIII, are marginally reduced, maybe due to increased consumption. At the same time, TFPI-1 levels and soluble thrombomodulin’s activity are proven to be increased. TFPI-2, which primarily derives from the placenta, is found to decrease due to the altered and abnormal placental development [3]. The significant reduction in the TFPI/TF ratio found in PE patients indicates that the elevated TFPI levels cannot compensate for the higher increase in TF [2,3,7,24,68].

### 3.6. Platelets

Regarding platelets, thrombocytopenia often characterizes PE and is dependent on the severity of the disease, and the total platelet count decreases as the severity of the disease progresses; the lower the platelet count, the higher the hazard for maternal and neonatal morbidity and mortality. As expected, thrombocytopenia with a platelet count of <100 × 10^9^/L is met in severe PE. Depending on PE severity, the platelet count is gradually restored after delivery within 3–5 days. regarding hemolysis, elevated liver enzymes, and low platelets (HELLP) syndrome, the platelet count’s fall may persist even after delivery [2,69,70]. MPV is likewise increased due to the release of more immature younger platelets, while thrombopoietin levels are elevated, reflecting a counteracting effort to produce more platelets [71,72].

During PE, the activity of platelets is elevated, marked especially by increased α-degranulation, resulting in the release of β-thromboglobulin and factor 4 and higher platelet clearance. P-selectin expression, platelet-bound and platelet-bindable immunoglobulins, and platelet-monocyte aggregates are significantly elevated in PE compared with normal pregnancies, indicative of platelet surface alterations and the platelet activation [2,69,70]. Platelet activation leads to “exhaustion” as immunological mechanisms increase platelet deposition at sites with endothelial impairment. Thus, platelet aggregation is lower when assessed by in vitro assessments [73,74].

## 4. Neonates

### 4.1. Neonatal Developmental Hemostasis

Age-related differences in coagulation components in healthy full-term newborns and children up to 16 years of age have been well established [75]. Overall, coagulation homeostasis in healthy newborns during the first days of life is achieved through maintaining balanced plasma procoagulant protein levels, thrombin production, and fibrinolysis capability [75].

#### 4.1.1. Neonatal Endothelium

The endothelium’s antithrombotic and fibrinolytic functions are age-dependent; hence, the neonatal endothelium is expected to differ from that of adults. In particular, endothelial cells’ selectin expression is indeed age-dependent. Around the 11th and 32nd gestational weeks, P-selectin and E-selectin levels reach those of adults, respectively [75,76]. Newborns’ endothelial cells have a limited capacity to counteract oxidative agents. Meanwhile, different gestational and postnatal ages have been found to have lower quantities of molecules with adhesion qualities [77].

#### 4.1.2. Coagulation and Fibrinolysis

Beginning at 11 weeks of gestation, coagulation factors are created by the fetus, and as the pregnancy develops and throughout postnatal life, their levels are seen to rise. Hence, preterm newborns have lower coagulation factors than older people and adults [78]. After birth, the vitamin K-dependent factors are around 30–50% of adult levels in preterm and full-term neonates and approach adult levels by about 6 months. In contrast, factors V, VIII, and XIII levels and vWF levels are nearly normal in newborns [79,80,81]. The levels of natural anticoagulants like AT, HCII, and protein C and S are lower in both preterm and full-term neonates, reaching about 50% at birth of what they are in adults. α-macroglobulin, on the other hand, is significantly higher. For full-term and preterm newborns, these coagulation inhibitors gradually rise to adult levels after 3 and 6 months. Preterm newborns have decreased plasmin/plasminogen system activity, decreased fibrinolysis, and a quicker thrombin production capacity to maintain the neonatal hemostasis balance [82].

#### 4.1.3. Platelet Function

In the first trimester, fetal platelets, arising from the fetal liver’s megakaryocytes, already account for around 150 × 10^9^/L. Around the 22nd to 24th gestational weeks, fetal platelets, originating mainly from the fetus’ bone marrow’s megakaryocytes, level at approximately 250 × 10^9^/L until delivery in full-term pregnancy (37th gestational week) [83]. Furthermore, newborns are characterized by platelet hypoactivity during the first 10 days of life, even though neonatal platelet levels are comparable with those of adults. This is due to weaker degranulation and less dense granules, which make it harder for platelets to bind to fibrinogen. Compared with adult platelets, neonatal platelets’ activation and aggregation are significantly impaired. It is worth noting that this impairment is found to be more severe in preterm newborns [84,85]. Newborn platelets express fewer α2-adrenergic receptors and protease-activated receptors, while thromboxane receptor signaling is downregulated. The impact and action of prostaglandin E1 are age-dependent. Intriguingly, the neonatal hemostatic balance is preserved due to counteracting parameters, such as a raised hematocrit and elevated vWf levels at birth, along with a slightly raised mean platelet volume (MPV), promoting the platelet–vessel interaction [75].

### 4.2. Hemostasis in Neonates Born to Pre-Eclamptic Mothers

It is essential to note that coagulation components do not cross the placenta. Thus, any perturbation of the hemostatic balance of the fetus and the newborn depends on the level of endothelial mediators causing endothelial dysfunction of the fetus/newborn born to mothers with PE. It is not directly linked to the hemostatic imbalance of the mother. This hypothesis presupposes that those fetuses/newborns from mothers with pre-eclampsia present an analogous endothelial dysfunction. The hemostatic link between mothers with PE and fetuses/neonates lies in the fact that PE is a placenta-mediated disease. The primary mediators leading to endothelial dysfunction and thus PE in mothers, such as cytokines, do not cross the placenta. However, the cells of the chorionic villi are in contact with the fetal circulation, and in placenta-mediated diseases with endothelial dysfunction, chorionic villi’s cells produce such cytokines, which can likewise insert the fetal circulation [86,87,88]. Thus, fetuses and newborns born to mothers with PE may experience a storm of mediators deriving from the placenta, which can concomitantly lead to an analogous fetal/neonatal endothelial dysfunction and thus to hemostatic perturbances. The notion of endothelial dysfunction in neonates born to mothers with PE is likewise supported by the fact that fetal plasma samples presented miRNA C19MC cluster alterations analogous to those in maternal plasma. The above suggests the placental expression of C19MC miRNAs and their release into maternal and fetal components and thus their potential circulation in fetal organs [89]. Another study showed similar results: C19MC miRNAs had an analogous expression and variance in fetal and maternal plasma and higher placental expression, whereas C14MC miRNAs had analogous expression in the placenta and fetal plasma, which was elevated compared with the C14MC miRNAs expression in maternal plasma [90].

As already stated, EPC’s defective number and function in PE play a crucial role in developing endothelial dysfunction. Regarding neonates, fetal endothelial colony-forming cells (ECFCs) are decreased in cord blood neonates born to mothers with PE. ECFCs in the cord blood of infants born to mothers with PE showed reduced proliferation, formed fewer tubules, and migrated less than controls [91]. An altered miRNA profile was found in cord blood-derived EPCs among infants born to women with PE [92]. A dysregulated methylation profile of fetal ECFCs in the cord blood of infants born to mothers with PE was found, which may precede clinical manifestations of PE [93]. In PE cases, the ability of fetal cord blood EPCs to incorporate into fetal endothelial cell networks is decreased [94].

Hemostatic imbalance and dysfunction in neonates born to mothers with PE have not been thoroughly studied. Thus, safe and concrete results have not been established. Raised levels of factors V and VIII and reduced levels of II, VII, and XI in cord blood have been reported [3,95]. Fibrinogen levels are controversial as they are either found to be reduced with high PAI-1 levels [96,97,98] or elevated [95]. Other studies assessing hemostatic parameters in neonates born to mothers with PE showed no statistical significance between those born to mothers with PE and those born to healthy mothers regarding fibrinogen, plasminogen, α2-macroglobulin, ATIII, t-PA, u-PA, PAI-1, PAI-2, and D-dimers [3,24]. As expected, those differences were statistically significant between mothers and neonates, with the neonates showing lower levels of the above parameters [24].

Regarding platelets, it is suggested that fetuses born to mothers with PE do not develop thrombocytopenia [74]. However, some studies noted that maternal PE is associated with thrombocytopenia, which lasts even until the 10th day of life and is rarely severe (<50 × 10^9^/L) [99,100,101]. The latter may be attributed to PE-associated placental ischemia, resulting in a counteracting increase in fetal erythropoietin, which subsequently suppresses fetal megakaryocytes’ series and propagation without excessive platelet consumption. The above results concerned small-for-gestational age neonates born to mothers with PE [101]. It is also suggested that this thrombocytopenia may be due to an increased platelet activation mediated by cytokines, such as IL-6 [99,100]. Nevertheless, it is essential to note that in case of severe early-onset PE, neonates are usually born prematurely. Prematurity is associated with a more immature immune system, and these neonates are more prone to neonatal sepsis, which can cause thrombocytopenia. Thus, in the case of thrombocytopenia occurring at birth among neonates born to mothers with severe early-onset PE, the potential presence of early-onset neonatal sepsis must be investigated [41].

A significant limitation of these studies is the type of sample used for assessment. Usually, the sample constitutes whole cord blood from umbilical veins during delivery, thus influencing the reliability of the results as cord blood flow differs from the normal blood flow noted in peripheral veins [24]. Likewise, this type of sample reflects more on the hemostasis of the placenta rather than neonatal hemostasis. It is essential to note that even if the sampling is conducted shortly after the cord is clamped, such techniques are associated with venous stasis, which can easily and quickly alter the coagulation parameters and affect the tests’ results [24,98].

## 5. Evaluation of Hemostasis in Healthy Pregnant Women and in Women with Pre-Eclampsia

### 5.1. Conventional Coagulation Tests (CCTs)

In healthy pregnancies, CCTs do not significantly differ. In the third trimester, a shortened activated partial thromboplastin time (APTT) may be present, and fibrinogen is mostly elevated [102,103,104].

Regarding pregnancies complicated with PE, some studies report little to no differences [4,7]. Nevertheless, most studies report an increase or decrease in APTT, prolonged prothrombin time (PT), elevated fibrinogen and D-dimers levels, and reduced AT activity in women with PE compared with healthy pregnant women [6,65,105]. Especially in severe forms of the disease and when the platelet count is lower than 100 × 10^9^/L, the hemostatic abnormalities are more eminently reflected in PT, APTT, and fibrinogen values [8]. This may be attributed to the fact that the propagation phase of coagulation among PE patients is more altered than the initiation phase.

The CCT results of several studies on healthy pregnant women and women with PE are shown in Table 1.

CCTs cannot sufficiently depict the alterations of the coagulation system [5]. This is because they are performed on platelet-poor plasma. In vivo clotting occurs on cell surfaces rather than in plasma. Thus, they cannot consider the dynamic interaction between platelets and factors of the coagulation cascade, coagulation inhibitors, and proteins of the fibrinolysis pathway [6,7]. Therefore, CCTs are unable to reflect the coagulation status of PE patients and thus cannot monitor the development of the disease. CCTs cannot distinguish between the physiological hypercoagulability of a healthy gestation and the excessive pathological hypercoagulability of PE, failing to reflect elements regarding the quality of the formed clot [4].

### 5.2. Viscoelastic Tests

Viscoelastic tests, such as TEG and ROTEM, requiring only a small blood sample, have the power to evaluate in a dynamic, universal, and coherent way the hemostatic profile of an individual. They monitor the process from the initiation of the coagulation cascade to the creation of a cross-linked fibrin clot and then its retraction and lysis by measuring the interaction between platelets, coagulation factors, fibrinogen, and the fibrinolysis pathway. They are easy to use, rapid, reproducible, and effective bedside methods [8,9,10]. They are primarily used in cardiac surgery, trauma, liver transplantation, and obstetric anesthesiology [9]. Viscoelastic tests use fully automated systems that work with cartridges, allowing for specific and predefined activators/reagents’ concentrations. Thus, they have become better standardized compared with the past [109,110].

Viscoelastic tests have limitations associated with assay and reagent composition and limitations associated with the measuring principle. Those associated with assay and reagent composition involve the heparin-like effect, the hyperfibrinolysis, and the direct oral anticoagulant effect quantification. Thus, results without heparin inhibitors can be altered by a heparin-like effect, while whether or not viscoelastic tests can detect low-grade hyperfibrinolysis is debated. Parallelly, the hemostatic assessment of patients receiving direct oral anticoagulants, direct thrombin inhibitors, or warfarin requires specialized reagents. The limitations associated with the measuring principle involve von Willebrand disease, platelet dysfunction, and the endothelium. The inherent lack of contact with collagen and the low shear stress makes viscoelastic tests non-sensitive to von Willebrand disease. Regarding platelets, platelet thrombin receptors can be stimulated by thrombin, which is generated in large amounts in standard assays. Therefore, viscoelastic tests are not sensitive to the effects of antiplatelet drugs. The endothelial components of hemostasis cannot be assessed as the endothelial fraction of thrombomodulin cannot be detected [109,110]. Finally, viscoelastic tests cannot identify individual coagulation factors, and the samples must be processed as soon as they are drained [8,9,10].

TEG and ROTEM parameters, their definitions and interpretations, and ROTEM assays are described in Table 2.

#### 5.2.1. TEG/ROTEM in Healthy Pregnancies

Regarding TEG parameters, reaction time (R) and clot kinetics (K) values and the lysis index at 60 min (LY60) are significantly decreased, and the maximum amplitude (MA), A-angle, and coagulation index (CI) values are significantly increased in healthy pregnant women compared with non-pregnant individuals. Functional fibrinogen TEG, which evaluates the contribution of fibrinogen to clot strength absent of any platelet function, shows a similar tendency: MA is higher among healthy pregnant women compared with non-pregnant controls [114]. The above results reflect a hypercoagulable state, with more stable fibrin clots and enhanced fibrinolysis [114,115,116,117].

Regarding ROTEM, the extrinsic (EXTEM) assay reveals a hypercoagulable profile of healthy pregnant women, as expected. The values for maximum clot firmness (MCF) and amplitude at 30 min (A30) significantly increase in all ROTEM assays, reflecting the formation of a stable and firm clot and slower fibrinolysis. The clotting time (CT) and clot formation time (CFT) are significantly decreased in the EXTEM and intrinsic (INTEM) assays, respectively, revealing a faster onset of clot formation affecting both the extrinsic and intrinsic pathways [104,118]. The MCF gradually and significantly increases as the gestation progresses from the first to the second and third trimesters (EXTEM assay) and from the second to third trimesters (INTEM and fibrinogen-FIBTEM assay) [103,118]. During the third trimester, the maximum lysis (ML) percentage is significantly reduced (INTEM and FIBTEM assays), reflecting reduced fibrinolysis. The MCF significantly correlates with fibrinogen levels as gestation progresses, especially in the EXTEM and FIBTEM assays [102]. A decrease in CT was apparent early in pregnancy [118], while early amplitude variables were significantly elevated during the second and third trimesters, especially in the FIBTEM assay [103]. There were no significant differences in any ROTEM parameters between labor and 1 h after placenta delivery [106].

Thus, pregnancies are characterized by hypercoagulation, with stable clot formation. TEG/ROTEM results are contradictory regarding fibrinolysis during healthy pregnancy. Clot formation initiation is not shortened, but once the clot formation is initiated, it progresses rapidly (stable CT and lower CFT in INTEM and EXTEM assays). These alterations gradually take place from the first trimester to the third. Table 3 summarizes the TEG/ROTEM results in healthy pregnancies.

Some studies have proposed TEG/ROTEM reference ranges for healthy pregnant women. However, these reference ranges have not been validated or accepted [117]. Table 4 shows these suggested reference ranges.

The main limitations of the above studies concern the sample size, of which some (three out of eight) are small (below 120 participants per group) [102,115,118] for establishing reverence values [122]. The exclusion criteria used were different among the studies. Some studies enrolled only third-trimester pregnant women or women at term, while others did not use trimester-specific time points [104,115,118]. Generally, as the methods used are not standardized, the results are not easily generalizable. Most studies have not performed CCTs. Even if CCTs do not adequately reflect hemostasis during pregnancy, the inclusion of CCTs in these studies could help better establish the inability of CCTs to depict hemostatic alterations in pregnancy. Likewise, the application of CCTs could permit the correlation of TEG/ROTEM parameters with CCTs [104,115,116,118]. Some studies were not longitudinal, and different subjects were assessed at different time points [102,103].

**Table 4:** Studies enrolling healthy pregnant women and the relevant rotational thromboelastometry (ROTEM) and thromboelastography (TEG) suggested reference ranges for this population. Values in bold and with an asterisk (*) indicate statistical significance at the level of α = 0.05 between healthy pregnant individuals and non-pregnant controls.

Rotational Thromboelastometry (ROTEM)											
Study	Trimester	Assay	CT (sec)	CFT (sec)	MCF (mm)	A-Angle (°)	A10 (mm)	A30 (mm)	LI30 (%)	ML (%)	Notes
Rheenen-Flach et al., 2013 [118]	Postpartum controls >6 weeks (19 women)	INTEM	127–262	44–88	57–69	71–81	-	56–68	-	-	Suggested reference ranges
		EXTEM	22–145	42–104	59–72	69–81	-	57–70	-	-	
	Healthy pregnant women 8–20 GW (45 women)	INTEM	119–213 *	40–86	59–72 *	73–82	-	57–71 *	-	-	
		EXTEM	38–97 *	45–101	60–75 *	68–82	-	59–75 *	-	-	
	Healthy pregnant women 20–32 GW (41 women)	INTEM	122–215	41–75 *	62–73 *	75–82 *	-	60–72 *	-	-	
		EXTEM	29–113	41–95	63–77 *	72–81 *	-	61–76 *	-	-	
	Healthy pregnant women 32–42 GW (44 women)	INTEM	107–213	36–70 *	63–78 *	76–82 *	-	62–77 *	-	-	
		EXTEM	3–180 *	31–101	64–79 *	72–83	-	63–78 *	-	-	
De Lange et al., 2014 [106]	Healthy pregnant women during labor (161 women)	INTEM	109–225	40–103	63–78	70–82	55–72	-	-	0–15	Suggested reference ranges
		EXTEM	31–63	41–120	42–78	67–83	48–74	-	-	0–41	
		FIBTEM	1–79	-	13–45	50–83	12–38	-	-	0–6	
		APTEM	33–62	42–118	61–79	69–82	54–72	-	-	0–15	
	Healthy pregnant women 1 h after delivery (161 women)	INTEM	98–225	37–118	48–78	67–82	46–73	-	-	0–15	
		EXTEM	34–66	44–154	55–78	63–81	44–73	-	-	0–44	
		FIBTEM	31–59	-	12–42	65–83	12–44	-	-	0–10	
		APTEM	31–71	47–158	56–78	60–81	43–72	-	-	0–14	
Lee et al., 2020 a [104] Lee et al., 2020 b [119]	Non-pregnant controls (132 women)	INTEM	115–245	42–103	59–76	70–82	-	59–76	-	-	Suggested reference ranges
		EXTEM	43–69	43–108	60–78	69–82	-	60–78	-	-	
		FIBTEM	40–74	-	16–34	67–81	-	16–34	-	-	
	Healthy pregnant women Term >37 GW (121 women)	INTEM	118–222	36–89 *	61–78 *	72–82	54–73	61–78 *	-	-	
		EXTEM	40–65 *	41–93	63–77 *	71–82	56–74	63–77 *	-	-	
		FIBTEM	41–66	-	16–40 *	69–81	15–37	16–40 *	-	-	
Shamshirsaz et al., 2021 [102]	Non-pregnant controls (33 women)	INTEM	131–220	50–93	57–72	71–79	57–72	-	-	2–13	Suggested reference ranges
		EXTEM	42–75	47–99	59–74	70–81	52–70	-	-	3–13	
		FIBTEM	40–64	-	10–31	63–82	10–32	-	-	0–13	
	Healthy pregnant women 1st trimester (34 women)	INTEM	152–203	45–91	57–72	72–81	52–69	-	-	2–13	
		EXTEM	45–75	44–97	61–76 *	71–81	54–72	-	-	3–17 *	
		FIBTEM	41–69	-	12–35	68–81	11–33	-	-	0–8	
	Healthy pregnant women 2nd trimester (34 women)	INTEM	137–207	43–91	57–74	71–81	51–71	-	-	2–15	
		EXTEM	44–69	42–100	60–77 *	71–81	52–74	-	-	2–17 *	
		FIBTEM	38–64	-	12–34	67–81	12–32	-	-	0–3 *	
	Healthy pregnant women 3rd trimester (41 women)	INTEM	127–207	42–88 *	61–77 *	73–81 *	53–72 *	-	-	2–10 *	
		EXTEM	40–71	38–94 *	63–79 *	71–82 *	54–75 *	-	-	2–12	
		FIBTEM	36–65	-	17–36 *	69–82 *	16–34 *	-	-	0–2 *	
Getrajdman et al., 2021 [120]	Healthy pregnant women at term (120)	NATEM	32–759	69–243	57–77	50–77	44–69	-	100–100	-	Suggested reference ranges
		NaHEPTEM	224–717	66–210	58–74	53–77	44–67	-	99–100	-	
**Thromboelastography (TEG)**											
**Study**	**Trimester**	**Assay**	**R (min)**	**K (min)**	**MA (mm)**	**A-angle (°)**	**LY30 (%)**	**LY60 (%)**	**CI**	**Notes**	
Polak et al., 2011 [115]	Non-pregnant controls (43 women)	Kaolin	4–8	1–4	55–73	47–74	-	0–15	−3–3	Suggested reference ranges	
	Healthy pregnant women 3rd trimester (60 women)		2–8 *	1–3 *	64–76 *	60–77 *	-	0–3 *	0–5 *		
Yang et al., 2019 [116]	Non-pregnant controls (145 women)	Kaolin	4.1–10.6	1.1–5.2	46.3–65.2	38.4–76.4	0–10.6	-	−7.5–1.9	Suggested reference ranges	
	Healthy pregnant women 1st trimester (252 women)		1.1–10.4 *	0.9–3.1	46.1–69.8 *	53.6–75.9	0–10.7	-	−5.5–2.5		
	Healthy pregnant women 2nd trimester (340 women)		3.9–9.7 *	0.8–2.4 *	49.8–72.1 *	56.7–78.0 *	0–9.7	-	−3.7–2.9 *		
	Healthy pregnant women 3rd trimester (161 women)		3.8–9.0 *	0.8–2.5 *	49.4–75.9 *	57.6–79.3 *	0–8.8	-	−3.0–2.6 *		
Xie et al., 2021 [123]	Healthy pregnant women 3rd trimester (125 women)	Kaolin	4–7.7	1.2–3.2	51.9–70.1	41.4–74.4	-	-	-	Suggested reference ranges	

CT: Clotting time (time to 2 mm amplitude); CFT: clot formation time (time from 2 mm to 20 mm); MCF: maximum clot firmness; A-angle (ROTEM): slope of tangent at 2 mm amplitude; A10: amplitude at 10 min; A30: amplitude at 30 min; LI30: lysis index at 30 min; ML: maximum lysis; INTEM: intrinsic thrombelastometry; EXTEM: extrinsic thromboelastometry; FIBTEM: fibrinogen thromboelastometry; NATEM: non-activated thromboelastometry; NaHEPTEM: non-activated thromboelastometry with heparinase; R: reaction time (time to 2 mm amplitude); K: clot kinetics (time from 2 mm to 20 mm); MA: maximum amplitude; A-angle (TEG): slope between R and K; LY30: percentage of the amplitude reduction 30 min after reaching the maximum amplitude; LY60: percentage of the amplitude reduction 60 min after reaching the maximum amplitude; CI: coagulation index; GW: gestational weeks; * With a mean/median statistically significant at the level of α = 0.05 between pregnant individuals and non-pregnant controls; -: Data not reported.

#### 5.2.2. TEG/ROTEM in Pre-Eclampsia

Regarding TEG, the results are controversial, showing either a hypercoagulable or hypocoagulable profile. However, this controversy is due to differences concerning the PE severity of the enrolled patients in each study. R and K were significantly lower in some cases, and the A-angle, MA, and CI were significantly higher in PE patients, reflecting a PE-associated hypercoagulable profile [4,6]. Likewise, the above results are statistically significant. They have a similar trend when comparing patients with mild PE with those with severe PE but with a platelet count over 100 × 10^9^/L, suggesting that as the severity of the disease progresses, the hypercoagulable state significantly increases given that thrombocytopenia is not severe [4]. The MA levels are significantly higher in mild PE, but as PE progresses to severe forms, the MA is significantly reduced compared with healthy pregnant women [124]. On the contrary, other studies reported mean values of R, K, A-angle, MA, and LY60 within the normotensive healthy pregnancy’s reference ranges [5,125]. However, even if not significant, the mean values for R and K were higher, and the mean values of the A-angle, MA, and LY60 were lower among women with PE, this time reflecting a hypocoagulable state [5,124]. Other studies have similar results, but with statistical significance; K was significantly increased in PE patients compared with healthy pregnant controls [65,123,124]. The more hypocoagulable profile noted in these cases is attributed to a vast consumption of clotting factors in PE patients with an essential parallel increase in TF. Parallelly, these results concern patients with severe forms of the disease, where severe thrombocytopenia is more eminent. Platelet phospholipid is necessary for the activation of factor X and prothrombin, and in these studies, where the platelet count is low, the clotting cascade is affected, resulting in prolonged R and K. The latter is supported by a reduced MA found in severe forms of PE in the same studies, reflecting an altered platelet function [5,65,123]. LY30 values were not significantly altered in PE patients, suggesting that the alterations in PE, where ATIII and Protein C activity are decreased and PAI-1 is increased, are insufficient to perturb fibrinolysis significantly [6]. It is worth noting that patients with gestational hypertension had significantly increased K values when compared with healthy pregnant controls. They also presented a non-significant decrease in R and a non-significant increase in the MA and A-angle values. These results reflect a hyperactive state of coagulability, even in gestational hypertension [123].

Regarding the TEG parameters’ diagnostic efficacy, CI seems to be the one with the highest sensitivity and specificity in diagnosing PE, and K seems to be a high-sensitivity test for diagnosing between mild and severe PE, but it lacks specificity [4]. Other studies suggest that MA has a significant diagnostic efficacy for PE compared with R and the A-angle [123]. It is likewise suggested that CI values under 0 in early gestation (under 20 gestational weeks) may have a predictive value for developing severe PE [5].

Regarding ROTEM, the EXTEM assay is more influenced in women with PE compared with healthy pregnant women, reflecting a hypercoagulable profile (the CFT and ML are decreased, and the MCF and A-angle are increased) and an increased speed of the propagation stage. The latter is associated with a hemostatic imbalance related to alterations in the extrinsic pathway, initiated by an essential increase in the TF, with the formation of fibrin clots with higher stability and significant fibrinolysis perturbation. The increased speed in the propagation phase is probably due to the increase in TF expression and activity. This is also reflected in the FIBTEM assay, where the platelet participation in fibrin clot formation is inhibited (the MCF and A-angle are increased). A significant linear correlation was found between fibrinogen and MCF-FIBTEM values in women with PE. The ML parameter was significantly decreased in all ROTEM assays, confirming the fibrinolysis’ perturbation [105].

Thus, PE is associated with an excessive hypercoagulability that is analogous with the severity of the disease, characterized by higher-stability fibrin clots and perturbed fibrinolysis. Nevertheless, when the platelet count is below the limit of 100 × 10^9^/L, there is a significant turnover to a hypocoagulability state. When the platelet count overpasses 100 × 10^9^/L, the TEG/ROTEM parameters, especially MA/MCF, seem to plateau; thus, it is suggested that they became insensitive to platelet count alterations. Table 5 summarizes the TEG/ROTEM results in pregnant women with PE.

The main limitations of the studies assessing the use of TEG/ROTEM in PE patients concern the different exclusion criteria implemented and the small sample sizes. Small sizes result in either a lack of evidence regarding specific subgroups or in the inclusion of heterogeneous populations, thus failing to reach statistical power [4,5,6,105,123]. More importantly, some cases do not include women with severe thrombocytopenia (<100 × 10^9^/L). The exact gestational week of hemostasis assessment is not always reported, not all women are assessed at all studies’ time points (different subjects are assessed at different time points), or TEG/ROTEM tests are performed only during third trimester. The latter is problematic as it cannot universally depict the hemostasis alteration during PE’s progression [4,6,105,123]. Because the methods used are not standardized and lack specific and widely accepted reference ranges, they are merely generalizable.

## 6. Future Insights

This review reveals three issues. First, it is necessary to clarify whether neonates born to mothers with PE develop endothelial dysfunction perturbing their hemostatic balance. Second, the number of studies assessing PE with viscoelastic tests, especially with ROTEM, is small. Third, the literature lacks studies assessing the hemostasis of neonates born exclusively to PE patients.

### 6.1. Neonates Born to Mothers with Pre-Eclampsia May Develop Endothelial Dysfunction

The literature is controversial regarding endothelial dysfunction in neonates and its potential association with neonatal hemostatic alterations. The levels of cellular fibronectin, stimulating increased NO production, are elevated in the cord blood of neonates derived from PE patients, indicating potential fetal endothelial activation [126]. However, other studies suggest that factors inducing maternal endothelial dysfunction in PE are not operative in fetal circulation [127,128]. Because PE is placenta-mediated, and chorionic villi cells are in contact with the fetal circulation, endothelial dysfunction mediators may enter the fetal circulation [86,87,88]. Therefore, studies should concentrate on neonates exclusively born to mothers with PE and assess neonatal endothelial dysfunction mediator levels. The blood sample should not be drained from cord vessels as the blood flow is not analogous with the neonatal peripheral circulation. Likewise, the clamped cord provokes stasis, potentially affecting the results. Blood samples should be drained directly from the newborn as soon as it is born. This approach will indeed reflect the actual neonatal endothelial status. If taken later, the endothelial profile may have been altered.

### 6.2. Viscoelastic Tests May Be Helpful in Pre-Eclampsia Management

PE is caused by endothelial dysfunction and concomitant hemostatic malfunctioning. CCTs were unhelpful in detecting and monitoring hemostatic alterations in PE patients [7]. Thus, the idea that PE prediction, detection, and treatment could rely on hemostatic assays, such as viscoelastic assays, remains feasible [2]. In PE, placental trophoblasts, fetus’ villus interstitial cells, and vascular endothelial cells overexpress TF, which is released to the blood elements. Thus, alterations occurring early during pregnancy may be better detectable with TEG/ROTEM [5,105].

TEG/ROTEM may be used for PE prediction. Different PE predictive markers have been proposed: increased impedance uterine arteries blood flow during the first trimester, sFlt-1, sENg, and placental growth factor [129,130]. TEG/ROTEM, being easier to perform and rapid, may also be used early in gestation for predicting the probability of developing PE as hemostatic alterations in women who will develop PE occur early in pregnancy and precede clinical symptoms [6].

Likewise, TEG/ROTEM may help evaluate the syndrome’s severity as it is positively correlated with the degree of hypercoagulability. Even if TEG/ROTEM parameters do not reliably alter during gestational hypertensive disorders, as soon as alterations are observed, a transition from mild PE to severe PE or HELLP syndrome is revealed [2,4]. MA increments during early gestation were associated with poor perinatal outcomes and pregnancy losses [125]. They may contribute to identifying those PE patients with higher thrombotic risk, based on alterations in parameters during gestation, as well as those who could benefit from altered antithrombotic treatment [105]; these tests helped diagnose and treat hemorrhagic episodes in patients with HELLP syndrome where CCTs were unhelpful [7].

### 6.3. Viscoelastic Tests May Be Helpful in Neonates Born to Pre-Eclamptic Women

Regarding neonates, viscoelastic tests are used in neonatal departments and have become part of the clinical routine [131]. However, no studies have assessed the hemostasis of newborns derived exclusively from pre-eclamptic mothers with TEG/ROTEM using neonatal (and not cordial) blood samples. The execution of viscoelastic tests in such neonates, for whom endothelial dysfunction is probable, early after birth and throughout their hospitalization may be beneficial; it could provide early indices of hemostatic imbalance, which precede clinical manifestations. The TEG/ROTEM dynamic does not diminish due to the fact that the reference ranges are not well established for neonates (especially for premature ones) as mainly parameters’ alterations should be taken into consideration and not values at individual time points.

## 7. Conclusions

Normal gestation is described as a procoagulant state. As PE develops, the normal pregnancy’s hypercoagulable balance is disrupted, leading to platelet hyperactivation and excessive pathological hypercoagulability. Hemostasis in PE is characterized by enhanced thrombin generation with perturbed fibrinolytic activity and elevated inhibitor proteins. Hemostatic imbalance and dysfunction in neonates born to mothers with PE have not been thoroughly studied, and few studies have addressed the issue. It is worth noting that fetuses born to mothers with PE may develop thrombocytopenia.

CCTs can not sufficiently depict the alterations of the coagulation system. This is because they are plasma-based. TEG and ROTEM, requiring only a small blood sample, have the power to evaluate in a dynamic, universal, and coherent way the hemostatic profile. TEG/ROTEM reveal that healthy pregnancies are characterized by hypercoagulation, with stable clot formation. These alterations gradually take place from the first trimester to the third. Parallelly, compared with normal pregnancy, TEG/ROTEM in PE reveals an excessive hypercoagulability, analogous with the severity of the disease, characterized by higher-stability fibrin clots. Higher clot stability is attributed to elevated fibrinogen levels, perturbated-reduced fibrinolysis, and altered red cell membrane phospholipid configuration. Nevertheless, when the platelet count is below the limit of 100 × 10^9^/L, there is a significant turnover to a hypocoagulability state.

TEG/ROTEM parameters can be used as markers to distinguish between the excessive pathological hypercoagulability of PE and the physiological hypercoagulability of a healthy gestation. These parameters can, to some degree, reflect the disease’s severity and may be used mainly as monitoring markers and secondarily as predictive markers for the disease.

Finally, TEG/ROTEM may be beneficial for the early detection of hemostatic imbalances in neonates derived from PE mothers. Endothelial dysfunction is possible among these neonates. More studies should be conducted assessing endothelial dysfunction and TEG/ROTEM use among these neonates.

## Figures and Tables

**Table 1:** Studies assessing conventional coagulation tests in: (**A**) non-pregnant controls vs. healthy pregnant women; and (**B**) healthy pregnant women vs. pregnant women with pre-eclampsia. Values are shown as the “Mean ± SD” or the “Median (IQR)”. Values in bold and with an asterisk (*) indicate statistical significance at the level of α = 0.05.

(A) Non-PREGNANT CONTROLS vs. HEALTHY PREGNANT WOMEN								
Study	Population	Platelets (×10^9^/L)	PT (sec)	aPTT (sec)	Fibrinogen (mg/dL)	D-Dimers (μg/mL)	ATIII (%)	Notes
Huissoud et al., 2009 [103]	Non-pregnant controls (20 women)	234 (194–266)	15 (14–15)	32 (28–35)	330 (310–360)	-	-	Values are “Median” (IQR)
	Healthy pregnant women 1st trimester (17 women)	266 (233–302)	15 (14–15)	32 (28–35)	400 (370–430) *	-	-	
	Healthy pregnant women 2nd trimester (9 women)	202 (181–222)	14 (14–15)	31 (28–34)	460 (430–480) *	-	-	
	Healthy pregnant women 3rd trimester (58 women)	138 (119–152) *	14 (14–15)	30 (28–32)	500 (440–580) *	-	-	
De Lange et al., 2014 [106]	Healthy pregnant women during labor (161 women)	214 (179–257)	-	-	490 (440–580)	0.18 (0.11–026) *	-	Values are “Median” (IQR)
	Healthy pregnant women 1 h after delivery (161 women)	203 (171–246)	-	-	470 (410–540)	0.31 (0.19–0.47)	-	
Shamshirsaz et al., 2021 [102]	Non-pregnant controls (33 women)	221 (192–253)	13.1 (12.8–13.4)	30.6 (27.8–32.2)	321 (279–445)	0.26 (0.22–0.45)	-	Values are “Median” (IQR). ^†:^ Statistically significant between 1st and 2nd trimester. ^‡^: Statistically significant between 1st and 3rd trimester.
	Healthy pregnant women 1st trimester (34 women)	208 (172–246)	12.9 (12.8–13.3)	29.2 (27.3–30.8)	407 (369–458) *	0.37 (0.26–0.47) *	-	
	Healthy pregnant women 2nd trimester (34 women)	195 (165–224)	12.9 (12.5–13.1)	27.6 (26.7–29.2)	449 (385–497)	**0.53 (0.37–0.82) ^†^**	-	
	Healthy pregnant women 3rd trimester (41 women)	**171 (137–205) ^‡^**	12.9 (12.5–13.2)	**27.7 (26.5–28.9) ^‡^**	**470 (437–518) ^‡^**	**0.85 (0.65–1.24) ^‡^**	-	
**(B) HEALTHY PREGNANT CONTROLS vs. WOMEN WITH PRE-ECLAMPSIA**								
**Study**	**Population**	**Platelets (×10^9^/L)**	**PT (sec)**	**aPTT (sec)**	**Fibrinogen (mg/dL)**	**D-Dimers (μg/mL)**	**ATIII (%)**	**Notes**
Orlikowski et al., 1996 [7]	Healthy pregnant controls	-	10.2 ± 0.6	28.5 ± 3.1	530 ± 100	-	98 ± 10	Values are “Mean ± SD”
	Pregnant women with PE and Eclampsia (49 women)	199 ± 90	10.4 ± 0.9	24.9 ± 3.6	540 ± 140	-	83 ± 15	
Yin et al., 1998 [107]	Healthy pregnant controls (19 women)	-	-	-	367 ± 116	-	105.8 ± 14.2	Values are “Mean ± SD”
	Pregnant women with PE (30 women)	-	-	-	455 ± 129 *	**-**	86.6 ± 15.1 *	
Sharma et al., 1999 [108]	Healthy pregnant controls (52 women)	225 ± 58	-	-	-	-	-	Values are “Mean ± SD”. ^†^: Statistically significant between pregnant women with severe PE and platelet count < 100 × 10^9^/L; pregnant women with mild PE; and with severe PE and platelet count ≥ 100 × 10^9^/L.
	Pregnant women with mild PE (140 women)	230 ± 67	-	-	-	-	-	
	Pregnant women with severe PE and platelet count ≥ 100 × 10^9^/L (80 women)	220 ± 67	-	-	-	-	-	
	Pregnant women with severe PE and platelet count < 100 × 10^9^/L (34 women)	67 ± 17 *^†^	-	-	-	-	-	
Tanjung et al., 2005 [24]	Healthy pregnant controls (27 women)	-	-	-	499 ± 130	1.6 ± 0.0018	93.4 ± 12.1	Values are “Mean ± SD”
	Pregnant women with PE At term (32)	-	-	-	462 ± 150	2.316 ± 0.0034	88.5 ± 14.4	
	Pregnant women with PE Preterm (8)	-	-	-	564 ± 170	1.283 ± 0.0018	84 ± 10.2 *	
Davies et al., 2007 [65]	Healthy pregnant controls (93 women)	257 ± 89	13.2 ± 0.9	28.7 ± 1.3	440 ± 100	-	-	Values are “Mean ± SD”
	Pregnant women with mild PE (23 women)	230 ± 83	13.2 ± 0.9	30.2 ± 4.3	410 ± 100	-	-	
	Pregnant women with severe PE (27 women)	177 ± 81 *	13.3 ± 1.2	31.3 ± 3.4 *	400 ± 90	-	-	
Spieza et al., 2015 [105]	Healthy pregnant controls (60 women)	228 ± 54	-	28 ± 3	-	0.491 ± 0.399	-	Values are “Mean ± SD”
	Pregnant women with PE (30 women)	206 ± 54	-	26 ± 3 *	-	0.563 ± 0.49	-	
Lidan et al., 2019 [4]	Healthy pregnant controls (59 women)	238.63 ± 63.62	10.8 ± 0.75	26.92 ± 2.58	435 ± 99	1.43 ± 0.5	97.37 ± 14.6	Values are “Mean ± SD”
	Pregnant women with mild PE (32 women)	242.5 ± 64.81	10.77 ± 0.66	27.30 ± 3.11	422 ± 103	1.51 ± 0.46	90.05 ± 17.19	
	Pregnant women with severe PE (26 women)	231.23 ± 53.21	10.57 ± 0.62	26.15 ± 4.4	396 ± 99	1.68 ± 0.72	81.85 ± 18.97	

PT: Prothrombin time; aPTT: activated partial thromboplastin time; ATIII: antithrombin III; IQR: interquartile range; SD: standard deviation; PE: pre-eclampsia. * With a mean/median statistically significant at the level of α = 0.05. -: Data not reported.

**Table 2:** Thromboelastography (TEG) and rotational thromboelastometry (ROTEM) parameters, their definitions, and their interpretations. This table also depicts the different ROTEM assays, their activators, and the differences among them [109,110,111,112,113].

Description	TEG Parameter	ROTEM Parameter	Reflecting
Clotting time (time to 2 mm amplitude)	Reaction time (R) in minutes	Clotting time (CT) in seconds	Activation–initiation phase First fibrin formation, functional behavior of clotting factors, and their deficiencies Dependent on clotting factors May be affected by congenital or acquired coagulopathies or anticoagulant therapy
Clot kinetics (time from 2 mm to 20 mm amplitude)	Clot kinetics (K) in minutes	Clot formation time (CFT) in seconds	Amplification phase Speed of initial fibrin deposition and cross-linking Dependent mainly on fibrinogen and its activation and to a lesser extent on platelets
Alpha angle (clot strengthening)	A-angle in degrees (slope between R and K)	A-angle in degrees (slope between the baseline and a tangent to the clotting curve through the 2 mm point)	Propagation phase Maximal speed of thrombin generation, fibrin deposition, and cross-linking (clot growth and strengthening) Dependent mainly on fibrinogen and its activation and to a lesser extent on platelets
Maximum strength/amplitude of the clot	Maximum amplitude (MA) in mm	Maximum clot firmness (MCF) in mm	Termination phase The ultimate strength of the fibrin clot (the stability of the clot) Dependent on platelets and fibrin interacting via GPIIb/IIIA May be affected by thrombocytopenia, thrombocytopathy
Clot firmness (amplitude) at set time after clotting time	Amplitude at 10, 30 min, etc. (A 10, 30, etc.) in mm (post-MA)	Amplitude at 10, 30 min, etc. (A 10, 30, etc.) in mm (post-CT)	Fibrinolysis Depends on the presence of plasmin, plasminogen, and their activators
Ratio of the amplitude and MA/MCF at set time after reaching maximum amplitude and clotting time, respectively	Lysis index or clot lysis at 10, 30, etc., minutes (LY or CL 10, 30, etc.) in percentage (post-MA)	Lysis index or clot lysis at 10, 30, etc. (LI or CL 10, 30, etc.) in percentage (minutes post-CT)	Fibrinolysis
Calculated through a mathematical formula considering the relative contribution of R, K, A-angle, and MA	Coagulation index (CI)	-	Spherical coagulability assessment. The higher the CI, the more hypercoagulable is the individual. A hypercoagulable state is defined as CI greater than +3.0 and coagulopathy as CI less than −3.0.
Maximum lysis detected during the run time (difference between MCF and the lowest amplitude after MCF, described in terms of percentage of MCF)	-	Maximum lysis (ML) in percentage	Fibrinolysis
**ROTEM assays**			
**Assay**		**Reagent(s)**	**Reflecting**
Intrinsic thromboelastometry (INTEM)		Phospholipids Ellagic acid	Intrinsic pathway. Assessment of factors XII, XI, IX, VIII, X, V, II, I, platelets, and fibrinolysis.
Extrinsic thromboelastometry (EXTEM)		Tissue factor	Extrinsic pathway. Assessment of factors VII, X, V, II, I, platelets, and fibrinolysis.
Fibrinogen thromboelastometry (FIBTEM)		Tissue factor	Type of EXTEM. Cytochalasin D is added as a platelet inhibitor. Fibrinogen levels and fibrin polymerization can be assessed in a functional way.
Aprotinin thromboelastometry (APTEM)		Tissue factor	Type of EXTEM. Aprotinin or tranexamic acid are added to inhibit fibrinolysis.
Non-Activated thromboelastometry NATEM)		-	Theoretically, it reflects the in vivo profile of the individual.
**TEG assays**			
**Assay**		**Reagent(s)**	**Reflecting**
K-TEG		Kaolin	Intrinsic pathway
H-TEG		Kaolin/Heparinase	Compared with K-TEG to assess the heparin effect
R-TEG		Kaolin/Tissue factor	Extrinsic pathway. Clot is formed immediately. Rapid interpretation of amplitude. Assessment of platelets and fibrinogen
TEG-FF		Kaolin/Tissue factor/Abciximab (GPIIb/IIIa platelet receptor inhibitor)	Fibrin polymerization. Compared with K-TEG to assess the contribution of fibrinogen to clot strength absent of any platelet function.
TEG-PM		Reptilase/Factor XIIIa + kaolin or ADP or arachidonic acid	Percentage of maximal platelet contribution in the presence of ADP or arachidonic acid to assess for an antiplatelet effect.
Native TEG		Calcium	Tissue factor expression on monocytes.

TEG: Thromboelastography; ROTEM: thromboelastometry; K-TEG: kaolin TEG; H-TEG: heparinase TEG; R-TEG: rapid TEG; TEG-FF: TEG functional fibrinogen; TEG-PM: TEG platelet mapping; ADP: adenosine diphosphate; GPIIb/IIIa: glycoprotein IIb/IIIa.

**Table 3:** Studies enrolling healthy pregnant women and assessing their coagulation profile using rotational thromboelastometry (ROTEM) and thromboelastography (TEG). The table shows the relevant ROTEM and TEG results. Values are shown as the “Mean ± SD” or the “Median (IQR)”. Values in bold and with an asterisk (*) indicate statistical significance at the level of α = 0.05 between healthy pregnant individuals and non-pregnant controls.

Rotational Thromboelastometry (ROTEM)											
Study	Population	Assay	CT (sec)	CFT (sec)	MCF (mm)	A-Angle (°)	A10 (mm)	A30 (mm)	LI30 (%)	ML (%)	Notes
Huissoud et al., 2009 [103]	Non-pregnant controls (20 women)	INTEM	159 (138–189)	78 (65–98)	58 (54–62)	-	-	-	98 (98–100)	-	Values are “Median (IQR)”
		EXTEM	51 (45–55)	101 (88–121)	59 (57–62)	-	-	-	99 (99–100)	-	
		FIBTEM	51 (44–53)	-	13 (11–16)	-	-	-	-	-	
		APTEM	63 (56–67)	96 (78–116)	58 (56–62)	-	-	-	99 (97–99)	-	
	Healthy pregnant women 1st trimester (17 women)	INTEM	168 (143–198)	74 (62–82)	62 (59–64)	-	-	-	99 (98–99)	-	
		EXTEM	54 (51–61)	82 (66–94) *	62 (60–94)	-	-	-	99 (99–99)	-	
		FIBTEM	59 (52–65)	-	18 (15–23)	-	-	-	100 (100–100)	-	
		APTEM	65 (58–83)	84 (71–88)	64 (60–67)	-	-	-	99 (98–100)	-	
	Healthy pregnant women 2nd trimester (9 women)	INTEM	145 (131–168)	62 (58–66) *	66 (64–68) *	-	-	-	100 (99–100)	-	
		EXTEM	54 (51–63)	77 (70–78) *	67 (66–71) *	-	-	-	99 (99–100)	-	
		FIBTEM	61 (50–64)	-	19 (19–22) *	-	-	-	100 (100–100)	-	
		APTEM	58 (51–84)	66 (65–68)	67 (65–82)	-	-	-	100 (99–100)	-	
	Healthy pregnant women 3rd trimester (58 women)	INTEM	155 (132–186)	66 (58–78) *	66 (63–69) *	-	-	-	100 (99–100)	-	
		EXTEM	53 (47–62)	74 (66–89) *	67 (64–71) *	-	-	-	100 (99–100)	-	
		FIBTEM	52 (46–65)	-	19 (17–23) *	-	-	-	-	-	
		APTEM	57 (52–75)	74 (64–96)	67 (64–70)	-	-	-	100 (99–100)	-	
Rheenen-Flach et al., 2013 [118]	Postpartum controls >6 weeks (19 women)	INTEM	195 ± 34	66 ± 11	63 ± 3	77 ± 2	-	62 ± 3	-	-	Values are “Mean ± SD”
		EXTEM	83 ± 31	73 ± 16	65 ± 3	75 ± 3	-	64 ± 3	-	-	
	Healthy pregnant women 8–20 GW (45 women)	INTEM	166 ± 24 *	63 ± 12	65 ± 3 *	77 ± 2	-	64 ± 34 *	-	-	
		EXTEM	67 ± 15 *	73 ± 14	68 ± 3 *	75 ± 3	-	67 ± 3 *	-	-	
	Healthy pregnant women 20–32 GW (41 women)	INTEM	168 ± 24	58 ± 9 *	67 ± 3 *	78 ± 2 *	-	66 ± 3 *	-	-	
		EXTEM	71 ± 21	68 ± 14	70 ± 4 *	77 ± 2 *	-	69 ± 4 *	-	-	
	Healthy pregnant women 32–42 GW (44 women)	INTEM	160 ± 27	53 ± 9 *	71 ± 4 *	79 ± 3 *	-	70 ± 4 *	-	-	
		EXTEM	92 ± 45 *	66 ± 18	72 ± 4 *	77 ± 3	-	71 ± 4 *	-	-	
De Lange, 2014 [106]	Healthy pregnant women during labor (161 women)	INTEM	147 (138–164)	55 (49–63)	71 (69–74)	79 (77–80)	64 (62–67)	-	-	5 (2–8)	Values are “Median (IQR)”
		EXTEM	45 (41–50)	69 (62–81)	71 (69–74)	77 (74–79)	64 (61–68)	-	-	7 (4–12)	
		FIBTEM	39 (37–44)	-	25 (22–28)	79 (76–80)	22 (20–26)	-	-	0 (0–0.5)	
		APTEM	43 (39–48)	69 (59–78)	71 (69–74)	77 (75–79)	64 (60–67)	-	-	4 (2–8)	
	Healthy pregnant women 1 h after delivery (161 women)	INTEM	137 (127–155)	57 (50–69)	71 (67–74)	78 (76–80)	64 (60–67)	-	-	4 (2–8)	
		EXTEM	45 (40–49)	73 (63–86)	71 (68–74)	76 (74–79)	64 (60–67)	-	-	8 (3–12)	
		FIBTEM	39 (36–42)	-	24 (20–28)	78 (75–80)	21 (18–25)	-	-	0 (0–0)	
		APTEM	41 (38–45)	72 (64–85)	71 (67–73)	76 (74–78)	63 (59–66)	-	-	5 (2–8)	
Lee et al., 2020 a [104] Lee et al., 2020 b [119]	Non-pregnant controls (132 women)	INTEM	167.7 ± 32.06	64.2 ± 15.3	68.3 ± 4.43	77.1 ± 2.73	-	68.4 ± 4.55	-	-	Values are “Mean ± SD”
		EXTEM	53.7 ± 6.26	65.7 ± 15.2	70.2 ± 4.01	77.4 ± 2.65	-	70 ± 4.13	-	-	
		FIBTEM	53.4 ± 7.85	-	24.1 ± 4.68	75.5 ± 3.81	-	24.1 ± 4.6	-		
	Healthy pregnant women Term >37 GW (121 women)	INTEM	162.5 ± 26.19	60.49 ± 12.6 *	69.6 ± 3.77 *	77.7 ± 2.47	-	69.6 ± 3.79 *	-	-	
		EXTEM	52.2 ± 5.91*	62.6 ± 13.08	71.1 ± 3.36 *	77.6 ± 2.64	-	71 ± 3.32 *	-	-	
		FIBTEM	51.7 ± 6.21	-	25.8 ± 4.86 *	76.2 ± 2.93	-	25.8 ± 4.93 *	-	-	
Shamshirsaz et al., 2021 [102]	Non-pregnant controls (33 women)	INTEM	173 (163–192)	72 (63–81)	64 (62–67)	75 (74–77)	59 (56–63)	-	-	7 (5–9)	Values are “Median (IQR)”
		EXTEM	58 (54–65)	74 (64–84)	67 (64–70)	76 (73–77)	61 (57–64)	-	-	8 (7–10)	
		FIBTEM	52 (48–58)	-	20 (16–25)	72 (70–75)	21 (17–26)	-	-	5 (1–8)	
	Healthy pregnant women 1st trimester (34 women)	INTEM	180 (168–188)	66 (61–75)	64 (62–67)	77 (75–77)	61 (58–63)	-	-	7 (5–9)	
		EXTEM	58 (53–71)	69 (58–81)	69 (67–71) *	76 (74–78)	63 (60–66)	-	-	9 (8–13) *	
		FIBTEM	55 (50–60)	-	23 (19–26)	74 (72–77)	21 (18–24)	-	-	2 (1–5)	
	Healthy pregnant women 2nd trimester (34 women)	INTEM	173 (161–184)	67 (59–73)	**66 (63–69)**	76 (75–78)	60 (58–63)	-	-	**8 (7–9)**	
		EXTEM	57 (53–61)	68 (62–79)	69 (66–72) *	76 (74–78)	63 (60–67)	-	-	9 (7–11) *	
		FIBTEM	51 (47–55)	-	23 (19–26)	75 (72–76)	21 (18–24)	-	-	0 (0–1) *	
	Healthy pregnant women 3rd trimester (41 women)	INTEM	168 (156–178)	64 (59–71) *	69 (66–71) *	77 (75–78) *	63 (60–65) *	-	-	6 (4–7) *	
		EXTEM	55 (50–61)	68 (57–75) *	71 (68–73) *	78 (76–79) *	66 (61–88) *	-	-	7 (6–9)	
		FIBTEM	51 (45–56)	-	27 (24–30) *	77 (74–78) *	25 (22–27) *	-	-	0 (0–1) *	
Getrajdman et al., 2021 [120]	Healthy pregnant women at term (120)	NATEM	482.5 (415–552)	112 (97.8–133)	66 (63–70)	69 (64.8–71)	**56 (52–61)**	-	100 (100–100)	-	Values are “Median (IQR)”
		NaHEPTEM	493 (421–559)	120 (98.8–137)	65 (62–69)	67 (64–71)	**56 (52–60)**		100 (100–100)		
Lee et al., 2022 [121]	Healthy pregnant women Term >37 GW (75 women)	EXTEM	54.3 ± 6.1	62.8 ± 11.5	70.9 ± 3.3						Values are “Mean ± SD”
		FIBTEM	53.9 ± 7.7	-	24.9 ± 4.5	-	-	-	-	-	
**Thromboelastography (TEG)**											
**Study**	**Population**	**Assay**	**R (min)**	**K (min)**	**MA (mm)**	**A-angle (°)**	**LY30 (%)**	**LY60 (%)**	**CI**	**Notes**	
Polak et al., 2011 [115]	Non-pregnant controls (43 women)	Kaolin	7.81 ± 2.76	2.72 ± 0.94	63.06 ± 5.4	54.38 ± 11.48	-	4.83 ± 3.64	−1.91 ± 3.01	Values are “Mean ± SD”	
	Healthy pregnant women 3rd trimester (60 women)		4.75 ± 1.74 *	1.48 ± 0.45 *	71.33 ± 4.45 *	69.58 ± 5.52 *	-	1.15 ± 1.78 *	2.68 ± 1.79 *		
Shreeve et al., 2016 [117]	Healthy pregnant women <14 GW (32 women)	Kaolin	6.2 ± 1.3	1.6 ± 0.4	67.4 ± 3.9	66 ± 5.1	1.2 ± 1.7	3.6 ± 2.1	-	Values are “Mean ± SD” * Statistically significant between healthy pregnant women 37–42 GW and controls of the above group postnatally	
	Healthy pregnant women 14–28 GW (24 women)		6.1 ± 1.3	1.3 ± 0.3	70.4 ± 3.7	68.5 ± 6.7	1.4 ± 0.8	5.1 ± 1.5	-		
	Healthy pregnant women 28–42 GW (33 women)		6.1 ± 1	1.4 ± 0.3	70 ± 4	68 ± 6	0.1 ± 0.8	4.2 ± 1.9	-		
	Healthy pregnant women 37–42 GW (23 women)		6.2 ± 1.3	1.3 ± 0.3	72 ± 3.3	70.1 ± 4.6	0.2 ± 0.4 *	1.8 ± 1.4 *	-		
	Controls of the above group postnatally		5.8 ± 1.2	1.2 ± 0.6	72.7 ± 3.7	72.4 ± 7.2	0.6 ± 0.9	3.1 ± 1.9	-		

CT: Clotting time (time to 2 mm amplitude); CFT: clot formation time (time from 2 mm to 20 mm); MCF: maximum clot firmness; A-angle (ROTEM): slope of tangent at 2 mm amplitude; A10: amplitude at 10 min; A30: amplitude at 30 min; LI30: lysis index at 30 min; ML: maximum lysis; INTEM: intrinsic thrombelastometry; EXTEM: extrinsic thromboelastometry; FIBTEM: fibrinogen thromboelastometry; APTEM: aprotinin thromboelastometry; R: reaction time (time to 2 mm amplitude); K: clot kinetics (time from 2 mm to 20 mm); MA: maximum amplitude; A-angle (TEG): slope between R and K; LY30: percentage of the amplitude reduction 30 min after reaching the maximum amplitude; LY60: percentage of the amplitude reduction 60 min after reaching the maximum amplitude; CI: coagulation index; IQR: interquartile range; SD: standard deviation; GW: gestational weeks; * With a mean/median statistically significant at the level of α = 0.05 between pregnant individuals and non-pregnant controls; -: Data not reported.

**Table 5:** Studies enrolling pregnant women with pre-eclampsia vs. healthy pregnant controls and assessing their coagulation profile using rotational thromboelastometry (ROTEM) and thromboelastography (TEG). The table shows the relevant ROTEM and TEG results. Values are shown as “Mean ± SD” or “Median (IQR)”. Values in bold and with an asterisk (*) indicate statistical significance at the level of α = 0.05 between pregnant women with pre-eclampsia and healthy pregnant controls.

Rotational Thromboelastometry (ROTEM)											
Study	Population	Assay	CT (sec)	CFT (sec)	MCF (mm)	A-Angle (°)	A10 (mm)	A30 (mm)	LI30 (%)	ML (%)	Notes
Spieza et al., 2015 [105]	Healthy pregnant controls (60 women)	INTEM	153 ± 20	67 ± 14	65 ± 5	76 ± 3	-	-	-	11 ± 4	Values are “Mean ± SD
		EXTEM	47 ± 8	75 ± 14	66 ± 4	75 ± 4	-	-	-	11 ± 5	
		NATEM	463 ± 54	116 ± 26	61 ± 4	67 ± 5	-	-	-	11 ± 4	
		FIBTEM	-	-	21 ± 4	73 ± 4	-	-	-	-	
	Pregnant women with PE (30 women)	INTEM	154 ± 27	64 ± 12	71 ± 4 *	77 ± 2	-	-	-	2 ± 3 *	
		EXTEM	48 ± 9	62 ± 15 *	72 ± 5 *	78 ± 4 *	-	-	-	2 ± 3 *	
		NATEM	432 ± 89	130 ± 55	64 ± 6	67 ± 9	-	-	-	1 ± 3 *	
		FIBTEM	-	-	28 ± 7 *	76 ± 6 *	-	-	-	-	
**Thromboelastography (TEG)**											
**Study**	**Population**	**Assay**	**R (min)**	**K (min)**	**MA (mm)**	**A-angle (°)**	**LY30 (%)**	**LY60 (%)**	**CI**	**Notes**	
Orlikowski et al., 1996 [7]	Healthy pregnant controls	Kaolin	7.8 ± 0.9	3.3 ± 0.7	59.7 ± 3.5	-	-	-	-	Values are “Mean ± SD”	
	Pregnant women with PE and eclampsia (49 women)		6.8 ± 1	3.1 ± 0.8	61.7 ± 6.6	-	-	-	-		
Sharma et al., 1999 [108]	Healthy pregnant controls (52 women)	Kaolin	26.2 ± 7.5	10.2 ± 3.5	66.5 ± 7.1	43.1 ± 9.1	-	-	-	Values are “Mean ± SD”. ^†^: Statistically significant between pregnant women with severe PE and platelet count < 100 × 10^9^/L and pregnant women with mild PE, severe PE, and platelet count ≥ 100 × 10^9^/L. ^‡^: Statistically significant between pregnant women with mild PE and severe PE and healthy pregnant controls.	
	Pregnant women with mild PE (140 women)		29.5 ± 7.2	10.8 ± 3.7	**69.3 ± 7.3 ^‡^**	41.6 ± 9.8	-	-	-		
	Pregnant women with severe PE and platelet count ≥ 100 × 10^9^/L (80 women)		29.6 ± 7.6	11.6 ± 4.2	66.1 ± 6.9	39.9 ± 9.4	-	-	-		
	Pregnant women with severe PE and platelet count < 100 × 10^9^/L (34 women)		37.4 ± 9.9 *^†^	20.9 ± 7.4 *^†^	52.1 ± 10.6 *^†^	23.6 ± 9.1 *^†^	-	-	-		
Davies et al., 2007 [65]	Healthy pregnant controls (93 women)	Celite-activated	4.5 ± 1.8	1.4 ± 0.5	73 ± 5	70 ± 9	-	-	-	Values are “Mean ± SD”	
	Pregnant women with mild PE (23 women)		4.9 ± 1.9	1.4 ± 0.4	73 ± 5	71 ± 6	-	-	-		
	Pregnant women with severe PE (27 women)		5.2 ± 2.5	2 ± 1.7 *	71 ± 8	66 ± 11	-	-	-		
Bulbul et al., 2015 [125]	Healthy pregnant controls (31 women)	Kaolin	7.8 ± 3.7	2.6 ± 1.1	66.1 ± 5.6	57.4 ± 9.2	1.2 ± 1.6	3.1 ± 3.1	−1.7 ± 3.7	Values are “Mean ± SD”	
	Pregnant women with PE (49 women)		9.5 ± 3	2.9 ± 1.2	66.5 ± 6.3	54.6 ± 9.7	1.7 ± 2.3	3.5 ± 3.4	−2.7 ± 3.6		
Lidan et al., 2019 [4]	Healthy pregnant controls (59 women)	Kaolin	4.71 ± 0.97	1.52 ± 0.37	-	65.58 ± 6.08	-	-	1.17 ± 0.93	Values are “Mean ± SD”. ^†^: Statistically significant between pregnant women with mild PE and pregnant women with severe PE.	
	Pregnant women with mild PE (32 women)		4.17 ± 0.88 *	1.36 ± 0.38 *	-	68.76 ± 5.27 *	-	-	3.06 ± 0.58 *		
	Pregnant women with severe PE (26 women)		3.7 ± 0.5 *^†^	1.13 ± 0.19 *^†^	-	71.95 ± 2.77 *^†^	-	-	3.74 ± 0.92 *^†^		

CT: Clotting time (time to 2 mm amplitude); CFT: clot formation time (time from 2 mm to 20 mm); MCF: maximum clot firmness; A-angle (ROTEM): slope of tangent at 2 mm amplitude; A10: amplitude at 10 min; A30: amplitude at 30 min; LI30: lysis index at 30 min; INTEM: intrinsic thrombelastometry; EXTEM: extrinsic thromboelastometry; NATEM: non-activated thromboelastometry; FIBTEM: fibrinogen thromboelastometry; R: reaction time (time to 2 mm amplitude); K: clot kinetics (time from 2 mm to 20 mm); MA: maximum amplitude; A-angle (TEG): slope between R and K; LY30: percentage of the amplitude reduction 30 min after reaching the maximum amplitude; LY60: percentage of the amplitude reduction 60 min after reaching the maximum amplitude; CI: coagulation index; SD: standard deviation; PE: pre-eclampsia; * With a mean/median statistically significant at α = 0.05 between healthy pregnant individuals and patients with pre-eclampsia; -: Data not reported.
